# Surveillance of public water supply fluoridation from the perspective of municipal health department technicians: a cross-sectional study, Goiás, 2022–2023

**DOI:** 10.1590/S2237-96222026v35e20250444.en

**Published:** 2026-03-16

**Authors:** Higor Andrade de Oliveira Gonçalves, Leandro Brambilla Martorell, Maria do Carmo Matias Freire

**Affiliations:** 1 Universidade Federal de Goiás, Faculdade de Odontologia, Goiânia, GO, Brazil

**Keywords:** Water Quality, Environmental Health Surveillance, Fluoridation, Oral Health, Dental Caries, Calidad del Agua, Vigilancia Sanitaria Ambiental, Fluoruración, Salud Bucal, Caries dental

## Abstract

**Objective:**

To investigate public water supply fluoridation surveillance actions in Goiás from the perspective of municipal health department technicians.

**Methods:**

A descriptive cross-sectional study was conducted with technicians responsible for water quality surveillance in each municipality of Goiás with fluoridated water. The final sample comprised 169 participants who completed an electronic questionnaire between 2022 and 2023. Data were analyzed using descriptive statistics.

**Results:**

More than half had completed higher education (56.8%) and held permanent positions (58.6%). Most reported knowing the Ministry of Health regulations, either fully or partially (95.3%), and considered fluoride analysis very important or important (87.8%). Less than half (42.6%) rated their level of training as very good or good, and 56.8% knew the purpose of fluoride. Analysis of this parameter was carried out by 26 municipalities (15.4%), of which 65.4% entered data into the Water Quality Surveillance Information System for Human Consumption (Sisagua). The most frequent reasons for not performing fluoridation surveillance were the lack of requests from the State Health Department (36.1%), materials and equipment (36.1%), knowledge (27.8%), and trained professionals (22.5%).

**Conclusion:**

Fluoridation surveillance actions were not systematically carried out by most technicians in the surveyed municipalities. Several difficulties and weaknesses were identified, indicating the need for actions to support compliance with the national policy on water surveillance for human consumption.

## Resumen

**Objetivo:**

Investigar las acciones de vigilancia de la fluoración del agua de abastecimiento público en Goiás desde la perspectiva de los técnicos de las secretarías municipales de salud.

**Métodos:**

Estudio descriptivo transversal realizado con técnicos responsables de la vigilancia de la calidad del agua de cada municipio de Goiás con agua fluorada. La muestra final estuvo compuesta por 169 participantes que respondieron un cuestionario electrónico entre 2022 y 2023. Los datos se analizaron mediante estadística descriptiva.

**Resultados:**

Más de la mitad tenía educación superior completa (56,8%) y era funcionario por concurso (58,6%). La mayoría informó conocer total o parcialmente las normas del Ministerio de Salud (95,3%) y consideró el análisis de flúor muy importante o importante (87,8%). Menos de la mitad (42,6%) evaluó su nivel de capacitación como muy bueno o bueno, y el 56,8% conocía la finalidad del flúor. El análisis de este parámetro fue realizado por 26 municipios (15,4%), de los cuales el 65,4% ingresaba datos en el Sistema de Información de Vigilancia de la Calidad del Agua para Consumo Humano (Sisagua). Los motivos más frecuentes para no realizar la vigilancia de la fluoración fueron la falta de solicitud por parte de la Secretaría de Estado de Salud (36,1%) y la falta de material y equipos (36,1%), de conocimiento (27,8%) y de personal capacitado (22,5%).

**Conclusión:**

Las acciones de vigilancia de la fluoración no se realizaban de manera sistemática según la perspectiva de la mayoría de los técnicos de los municipios estudiados. Se señalaron diversas dificultades y debilidades, lo que indica la necesidad de acciones que apoyen el cumplimiento de lo previsto en la política nacional de vigilancia del agua para consumo humano.

**Aspectos éticos**

A presente pesquisa respeitou os princípios éticos, obtendo os seguintes dados de aprovação:

**Comitês de ética em pesquisa**: **Números dos pareceres**, **Datas de aprovação**

Universidade Federal de Goiás: 5.190.841, 31/12/2021

Secretaria de Estado de Saúde de Goiás: 5.249.236, 17/2/2022

Certificado de apresentação de apreciação ética: 53750321.9.0000.5083 53750321.9.3001.5082

**Registro do consentimento livre e esclarecido** : Obtido de todos os participantes.

## Introduction 

Oral diseases represent a major public health challenge at the global, national, and local levels. Dental caries persists at high levels in Brazil, despite the decline of its prevalence in the permanent dentition of children and adolescents (1). Public water supply fluoridation involves adjusting the fluoride ion concentration of water intended for human consumption. Due to its effectiveness, safety, broad coverage potential, and low cost, it is the primary collective method for caries prevention and is recommended by research institutions and health systems (2–4). 

Fluoridation is an approach adopted in different ways around the world. While in certain countries or regions this strategy is widely applied, in others its implementation is limited or nonexistent. In Brazil, fluoridation began in 1953 in Baixo Guandu, Espírito Santo. This public health measure was established by Law No. 6,050 of 24 May 1974. 

To ensure that fluoridation delivers the expected benefits and poses minimal risks to the population, it is essential to verify that fluoride concentrations comply with the parameters established for each region. Surveillance of this method helps prevent both deficient supply, which compromises caries prevention, and overdosing, which can cause adverse effects such as dental fluorosis (5). The implementation of fluoridation in the country and the maintenance of surveillance actions are provided for in the National Oral Health Policy (6), established by Federal Law No. 14,572 of 8 May 2023 (7). 

The control and surveillance of the quality of water intended for human consumption are carried out within the scope of the Brazilian Unified Health System by environmental surveillance agencies or organizations not directly responsible for system operation. These actions are part of the National Program for Surveillance of Water Quality for Human Consumption (*Programa Nacional de Vigilância da Qualidade da Água para Consumo Humano*, Vigiagua) and its corresponding Water Quality Surveillance Information System for Human Consumption (*Sistema de Informação de Vigilância da Qualidade da Água para Consumo Humano*, Sisagua) (8). 

The parameters for fluoride concentrations are determined by the Ministry of Health’s ordinances (9,10). The maximum concentration must be 1.5 mg/L or ppm, with possible variations between 0.6 and 1.7 mg/L, depending on the annual mean of daily maximum temperatures in each locality. 

Due to the lack of systematized information on the implementation of surveillance actions nationwide, scientific research can expand knowledge and support the application and evaluation of relevant policies. Studies with this purpose are scarce, although several publications report laboratory analyses of fluoride levels at the local level (11,12). In 2005, five of the 17 Brazilian capitals with fluoridation practiced fluoridation surveillance (13). In Brazilian municipalities with more than 50,000 inhabitants, inequalities in surveillance actions across the country’s five macro-regions were revealed in 2014 (5,14). In the Central-West region, 68.6% of the municipalities investigated had fluoridated water, and only the Federal District performed external control in 2014 (15). 

In Goiás, fluoridation began in 1985 and, by 2023, covered 78.9% of the municipalities. Two previous studies analyzed fluoride levels in water samples from municipalities in Goiás in 2011-2013 (16) and in 2019 (17). The surveillance actions for fluoridation across all fluoridated municipalities are unknown. There is, therefore, a concerning knowledge gap on this issue. In this regard, reports from professionals directly involved may help elucidate factors that facilitate or hinder municipalities’ surveillance performance (18).

This study aimed to investigate public water supply fluoridation surveillance actions in Goiás from the perspective of environmental surveillance technicians of municipal health departments. The results may be useful for revealing the current situation, supporting adjustments to the fluoridation surveillance strategy within municipalities and the State Health Department, and clarifying the national situation.

## Methods 

### Study design

This is a cross-sectional descriptive observational study. 

### Setting 

Goiás is the most populous state in the Central-West region and the 11^th^ in the country. Its population was 7,056,495 inhabitants, with more than 90% living in urban areas, according to the 2022 Census by the Brazilian Institute of Geography and Statistics (Instituto Brasileiro de Geografia e Estatística, IBGE). Of the 246 municipalities in Goiás, 194 (78.9%) had fluoridated water in 2022 and were included in the study. 

### Participants 

Participants were professionals (technicians and inspectors in environmental surveillance) from the respective municipal health departments responsible for monitoring the quality of public water supply in each municipality, totaling 194 individuals. The invitation to participate in the study was made by the Health Surveillance Superintendence of the State Health Department (*Superintendência de Vigilância em Saúde da Secretaria de Estado da Saúde*). To validate the inclusion criterion, the questionnaire included questions regarding the professionals’ responsibilities.

### Variables 

The variables investigated were related to the sociodemographic and work-related characteristics of the technicians; knowledge and perceptions about water fluoridation surveillance; knowledge about the purpose of fluoride; methodological aspects of water fluoridation surveillance carried out by the municipalities; and reasons for not performing this activity, as well as perspectives for its implementation. 

### Data sources and measurement

For data collection (June 2022 to June 2023), a self-administered electronic questionnaire with both closed- and open-ended questions was used via Google Forms. The researchers developed the instrument based on the study objectives and previous research (5,11–14). The first version was reviewed by two faculty members participating in the project team. It was then reviewed by managers from the Health Surveillance Superintendence of the State Health Department, responsible for public water supply quality surveillance, as well as by four faculty members in the field of collective oral health with expertise in research questionnaires. 

The next phase consisted of a pilot study conducted via an online meeting with technicians from five municipalities. The final version was administered to participants during an online meeting organized for this purpose and convened by the State Health Surveillance Superintendence. Municipal technicians who did not attend the meetings later received the questionnaire by email. 

### Statistical methods

The data collected through the questionnaires were automatically stored in a Microsoft Excel spreadsheet. Subsequently, the data were analyzed using descriptive statistics (number and percentage) with the Statistical Package for the Social Sciences (SPSS) version 28.0. Two researchers categorized responses to the open-ended questions through content analysis. The emerging categories were quantified and subjected to descriptive statistical analysis.

## Results 

The questionnaire was completed by all invited technicians (n=194), one from each fluoridated municipality, resulting in a 100.0% response rate. Of these, 25 technicians were excluded from the analysis because they reported not performing direct surveillance functions of the public water supply. Participants’ ages ranged from 19 to 63 years (mean 39.2; standard deviation 10.9) (Table 1). Slightly more than half were male (50.9%), had completed higher education with or without postgraduate studies (56.8%), held permanent positions (58.6%), and had been working for up to five years in the surveyed municipal health departments (56.2%). 

**Table 1:** Sociodemographic and work-related characteristics of water quality surveillance technicians. Fluoridated municipalities of Goiás, 2022–2023 (n=169)

Variables	n (%)
Sociodemographic	
**Age group** (years)	
19–30	39 (23.1)
31–40	54 (32.0)
41+	68 (40.2)
No response	8 (4.7)
Sex	
Female	82 (48.5)
Male	86 (50.9)
Other	1 (0.6)
**Education level**	
Elementary education	2 (1.2)
High school	71 (42.0)
Higher education	55 (32.5)
Higher education with postgraduate studies	41 (24.3)
**Related to work at the Municipal Health Department**	
**Current employment relationship**	
Civil servant	99 (58.6)
Contracted employee	70 (41.4)
**Length of employment** (years)	
Less than a year	23 (13.6)
1–5	72 (42.6)
6–10	34 (20.1)
11–20	36 (21.3)
21+	14 (8.3)

Most respondents reported partial knowledge of the Ministry of Health regulations (72.8%) and rated fluoride analysis as very important (56.2%) or important (31.4%) (Table 2). When asked about their level of training to perform water fluoridation surveillance, 18.3% rated it as very good and 24.3% as good. 

**Table 2:** Knowledge and perception of water quality surveillance technicians about water fluoridation surveillance. Fluoridated municipalities of Goiás, 2022–2023 (n=169)

Variables	n (%)
**Do you know the Ministry of Health regulations for water quality control and surveillance?**	
Yes, completely	38 (22.5)
Yes, partially	123 (72.8)
No	8 (4.7)
**How would you rate the importance of analyzing fluoride levels in the public water supply?**	
Very important	95 (56.2)
Important	53 (31.4)
Neutral	5 (3.0)
Less important	0
Unknown	16 (9.5)
**How would you rate your level of training to perform water fluoridation surveillance?**	
Very good	31 (18.3)
Good	41 (24.3)
Fair	49 (29.0)
Poor	19 (11.2)
Very poor	5 (3.0)
Unknown	24 (14.2)

Of the total 169 respondents, 56.8% reported knowing the purpose of fluoride in the public water supply (Table 3). Among the responses to the open-ended question about its purpose, the most frequent were related to oral health (82.3%). Within this category, 61.4% mentioned the prevention, reduction, or control of dental caries. The remaining responses referred to mouth or tooth health, general health, and water quality. 

**Table 3:** Knowledge of water quality surveillance technicians about the purpose of fluoride. Fluoridated municipalities of Goiás, 2022–2023 (n=169)

Variables	n (%)
**Do you know the purpose of fluoride in the public water supply?**	
Yes	96 (56.8)
No	73 (43.2)
**Purpose of fluoride** (**n=96)a**	
Oral health	79 (82.3)
Prevention/reduction/control of dental caries	58 (60.4)
Prevention of dental caries and fluorosis	1 (1.0)
Oral health	9 (9.4)
Formation/maintenance/strengthening/protection of teeth	9 (9.4)
Prevention of oral diseases	1 (1.0)
Elimination of certain bacteria in the mouth	1 (1.0)
General health	5 (5.2)
Health improvements	2 (2.1)
Disease prevention	2 (2.1)
Prevention and awareness	1 (1.0)
Water quality	7 (7.3)
Improve water quality	4 (4.2)
Assess water quality, if chlorine levels are high	1 (1.0)
Detect water purity	1 (1.0)
“Fluoride is poison.”	1 (1.0)
No response	5 (5.2)

^a^Open-ended question.

According to the technicians, 26 municipalities (15.4%) analyzed fluoride levels in the past five years, while 20.7% of technicians did not know how to answer (Table 4). Of the 26 municipalities that conducted the analysis, 88.5% did so monthly, and 46.2% used the SPADNS photometric method. The analyses were conducted mainly in municipal health departments and in the Central Public Health Laboratory, with the former predominating (69.2%). In the municipal health departments (n=18), analyses were mostly performed by trained professionals (88.9%) and were conducted under the supervision of the State Health Department (56.2%). Support from the State Health Department and its regional health offices was reported by 38.5% of municipalities. In 19.2% of cases, oral health teams from municipal health departments participated in fluoride surveillance activities. A total of 23.1% of technicians reported difficulties due to a lack of space, equipment, supplies, professional training, and vehicles for collecting water samples. The insertion of data regarding fluoride content in Sisagua was reported by 65.4% of respondents.

**Table 4:** Methodological aspects of water fluoridation surveillance carried out by municipalities, according to water quality surveillance technicians. Fluoridated municipalities of Goiás, 2022–2023

Variables	n (%)
**Fluoride level analysis conducted by the municipality in the last five years (n=169)**	
Yes	26 (15,4)
No	108 (63,9)
Unknown	35 (20,7)
**Frequency of analysis (n=26)**	
Monthly	23 (88,5)
Weekly	1 (3,8)
Annually	1 (3,8)
Unknown	1 (3,8)
**Method used (n=26)**	
Photometric (SPADNS)	12 (46,2)
Colorimetric (alizarin visual method)	6 (23,1)
Electrometric	3 (11,5)
Unknown	5 (19,2)
**Location of fluoride analysis (n=26)**	
Municipal Health Department with portable equipment	18 (69,2)
Central Public Health Laboratory	5 (19,2)
Unknown	3 (11,5)
**Training of professionals, in the case of municipal health departments (n=18)**	
Yes, by the State Health Department	10 (55,6)
Yes, by the company that provided the equipment	4 (22,2)
Yes, but do not know who conducted the training	2 (11,1)
No	2 (11,1)
**Support from the State Health Department and/or its regional health offices for fluoride analysis (n=26)**	
Yes	10 (38,5)
No	11 (42,3)
Unknown	5 (19,2)
**Participation of oral health professionals (n=26)**	
Yes, from the Municipal Health Department itself	5 (19,2)
No	16 (61,5)
Unknown	5 (19,2)
**Difficulties faced by the municipality in fluoride analysis (n=26)**	
Lack of transportation/vehicle for water collection	2 (7,7)
Delays in acquiring products and calibrating equipment	2 (7,7)
Lack of adequate space/equipment/professional training	2 (7,7)
None	18 (69,2)
Unknown	2 (7,7)
**Entry of surveillance data into the Water Quality Surveillance Information System for Human Consumption (Sisagua) (n=26)**	
Yes	17 (65,4)
No	9 (34,6)

Results were analyzed for the 108 municipalities that did not conduct fluoride surveillance during the study period (Table 5). The most frequent reasons for not performing this activity were the lack of a requirement/request from the State Health Department (36.1%), the lack of appropriate materials and equipment (36.1%), and the municipality’s lack of knowledge (27.8%). A total of 58.3% of respondents expressed the intention to initiate this activity in the future. More than one-third did not know how to answer or did not respond to the two previous questions. Among those who reported municipal interest in conducting surveillance in the future (n=63), 55.6% stated they would need support from the State Health Department for the acquisition of appropriate equipment and materials, technical assistance, and professional training/capacity building. 

**Table 5:** Reasons for not conducting fluoridation surveillance and prospects for its implementation, according to water quality surveillance technicians. Fluoridated municipalities of Goiás, 2022–2023 (n=108^a^)

Variables	n (%)
**Reasons for not conducting fluoride analysis^b^**	
Not mandatory/not requested by the State Health Department	39 (36,1)
Lack of adequate materials and equipment	30 (27,8)
The municipality lacks knowledge on how to conduct it	24 (22,5)
Lack of trained professionals	2 (1,9)
The water treatment company conducts the analyses	20 (18,5)
Unknown	18 (16,7)
No response	39 (36,1)
**Intention to conduct in the future**	
Yes	63 (58,3)
No	2 (1,9)
Unknown	14 (13,0)
No response	29 (26,8)
**Needs from the State Health Department^c^ (n=63)**	
Equipment and materials, technical support, and training/capacity building	35 (55,6)
Equipment and materials only	6 (9,5)
Capacity building only	17 (27,0)
Unspecified	5 (7,9)

^a^Only municipalities that did not conduct fluoride surveillance; ^b^ More than one response possible, not totaling 100.0%; ^c^ Open-ended question.

## Discussion 

This study aimed to examine public water supply fluoridation surveillance activities in Goiás from the perspective of environmental surveillance technicians at municipal health departments. The absence of fluoride concentration analysis in the public water supply in most fluoridated municipalities underscores a concerning gap in water quality surveillance, particularly given the importance of this measure for the population’s oral health (19). 

Only 26 municipalities (15.4%) included fluoride in their surveillance activities. Not all performed monthly analyses or recorded data in Sisagua, contrary to federal government standards (9,10). Among the 108 municipalities that did not conduct fluoride surveillance, 58.3% expressed interest in initiating this activity. This highlights the need to strengthen fluoridation surveillance strategies at both the municipal and state levels.

Low adherence to fluoride surveillance activities was also observed in municipalities in Goiás with more than 50,000 inhabitants (14,15). This study represents the first state-level investigation to encompass a wide range of variables. When interpreting the results, it is important to consider that they are based solely on information provided by technicians from each municipality. Therefore, the reports may not be entirely accurate or may not reflect what is actually practiced. The high percentage of missing responses to specific questions about fluoride may indicate a lower priority assigned to surveillance of this parameter compared to others that are formally agreed upon and jointly monitored with the State Health Department.

The limited participation of oral health teams in fluoride surveillance activities suggests a possible disconnection between oral health promotion strategies and water quality surveillance practices in the municipalities. These findings underscore the importance of including content related to fluoridation surveillance in undergraduate dentistry curricula, which could foster greater integration between oral health and surveillance teams within the framework of common risk factors for oral and systemic diseases (20).

The predominance of higher educational levels and civil service employment among respondents can be considered positive aspects for the performance of surveillance activities. Conversely, in a previous study about the pattern of organizational commitment observed among sanitary surveillance inspectors in municipalities of Goiás, greater affective commitment and a stronger sense of duty in job performance were found among older respondents, those with lower educational levels, and those without permanent employment ties to the municipal health departments (21).

A high percentage of respondents in the total sample considered fluoride analysis to be very important or important, indicating recognition of its relevance to the population’s oral health. However, fewer than half reported being satisfied with their level of training for this activity. Slightly more than half reported knowing the specific purpose of fluoride, of whom nearly 18% did not mention any oral health benefits. A single report from a technician who described fluoride as poison stands out, indicating misinformation about this substance, even among professionals in the Brazilian Unified Health System (*Sistema Único de Saúde*, SUS). 

There are concerning gaps in participants’ technical knowledge that may negatively affect the performance of their functions. Permanent health education strategies (22) based on high-quality scientific evidence are recommended so that technicians can perform their duties in accordance with legislation (9–10) and ensure the quality of the water provided to the population. As an example of such a strategy, the inclusion of specific modules on fluoridation (2,3) in training or continuing education within the National Program for Surveillance of Water Quality for Human Consumption (Vigiagua) (8) is noteworthy. 

The lack of materials, equipment, transportation, knowledge, and trained professionals, as reported, constitutes barriers to fluoride analysis by municipalities and represents a crucial issue to be addressed. Similar results were reported in a study that assessed the difficulties and limitations faced by technicians responsible for water quality surveillance in seven municipalities of the metropolitan region of Greater Vitória, Espírito Santo (25). The lack of essential resources can compromise municipalities’ ability to perform accurate and consistent analyses, undermining the effectiveness of water fluoridation. In this study, technicians from municipalities that did not conduct surveillance also also reported a lack of requirement or request from the State Health Department. 

The underutilization of Sisagua for recording fluoride surveillance data has been reported at both national and local levels (13,23–25). In this study, 65.4% of the 26 municipalities in Goiás that conducted fluoride surveillance reported entering their data into the system. This situation highlights the need for state health departments to monitor actions at the municipal level, identify inconsistencies and potential errors, and provide technical support when necessary.

Although public water supply fluoridation has been mandated by federal legislation since 1974 and is supported by robust scientific evidence of its effectiveness (3,4), surveillance of this method faces significant operational, structural, and political challenges in Brazilian municipalities. This reality reflects a recurring difficulty in the public health sector: the fragility of public policy implementation. The implementation stage is often undervalued and is characterized by barriers at the local level, including conflicts of interest, a lack of technical and human resources, and low political priority, which hinder the effective execution of national guidelines (26).

In the specific field of health surveillance, the consolidation of the national policy faces limitations, including insufficient information systems, limited intersectoral integration, and fragmentation between surveillance and primary care, which compromise the effectiveness of actions (27). These challenges are even more pronounced in policies such as fluoridation surveillance, which require intersectoral cooperation among sanitation, environmental health, and oral health sectors.

Regarding oral health, despite normative advances and the institutionalization of surveillance practices (6,7), challenges persist, including shortages of qualified professionals, weak local monitoring systems, and a lack of incentives for the continuity of actions (28). The Federative coordination of public health policies in Brazil still lacks effective mechanisms of induction and accountability among the Federated entities for the standardization of practices and compliance with established standards (29).

To understand the weak adherence to fluoridation surveillance, it is important to recognize that the mere existence of legal guidelines does not guarantee their implementation. Implementation is a complex political-institutional process that depends on technical capacity, local political support, and an adequate administrative structure to be carried out effectively. 

A major strength of this study is that it was conducted from the perspective of water surveillance workers within the Brazilian Unified Health System (SUS), who provided valuable insights. Other strengths included the sample’s representativeness at the state level and the scope of the variables investigated. 

The main limitations of the study were the generally low reliability of data obtained through electronic questionnaires (30) and the lack of reliability testing for the instrument. There was also the possibility of an opinion bias, which may have led to skewed responses and results that do not fully reflect the study population’s true opinions. 

Based on the reports from technicians in the surveyed municipalities, it can be concluded that fluoridation surveillance actions were not systematically carried out. Only 15.4% of municipalities conducted monitoring, and among these, 65.4% entered data into Sisagua. The main reasons for not conducting fluoride analysis were related to lack of knowledge and training (50.3%), lack of materials and equipment (36.1%), and the non-mandatory nature of the activity (36.1%). 

The difficulties and weaknesses identified indicate the need for actions at the municipal and state health department levels to support compliance with the provisions of the national water surveillance policy and related regulations (8–10). The inclusion of fluoride among the parameters to be monitored is essential to ensure the effectiveness and safety of fluoridation in preventing dental caries in the population (7). 

## Data Availability

The database and analysis codes used in this study will be made available upon request to the authors through the contact information provided in the Correspondence section.
