# Supplementary material for: Surveillance of public water supply fluoridation from the perspective of municipal health department technicians: a cross-sectional study, Goiás, 2022–2023
Source: Epidemiol Serv Saude. 2026 Mar 16;35:e20250444. doi: 10.1590/S2237-96222026v35e20250444.en (PMC12995270; doi:10.1590/S2237-96222026v35e20250444.en)
Supplement: Translation

# Translation: A vigilância da fluoretação da água de abastecimento público na perspectiva dos técnicos das secretarias municipais de saúde: estudo transversal, Goiás, 2022-2023

**DOI:** 10.1590/S2237-96222026v35e20250444.pt

**Authors:** Higor Andrade de Oliveira Gonçalves, Leandro Brambilla Martorell, Maria do Carmo Matias Freire

**Affiliations:** 1 Universidade Federal de Goiás, Faculdade de Odontologia, Goiânia, GO, Brasil

**Keywords:** Qualidade da Água, Vigilância Sanitária Ambiental, Fluoretação, Saúde Bucal, Cárie Dentária

## Abstract

**Objetivo:**

Investigar as ações de vigilância da fluoretação da água de abastecimento público em Goiás na perspectiva dos técnicos das secretarias municipais de saúde.

**Métodos:**

Estudo descritivo transversal realizado com técnicos responsáveis pela vigilância da qualidade da água de cada município goiano com água fluoretada. A amostra final foi composta por 169 participantes que responderam a um questionário eletrônico entre 2022 e 2023. Os dados foram analisados por meio de estatística descritiva.

**Resultados:**

Mais da metade tinha curso superior completo (56,8%) e era concursada (58,6%). A maioria relatou conhecer total ou parcialmente as normas do Ministério da Saúde (95,3%) e considerou a análise do fluoreto muito importante ou importante (87,8%). Menos da metade (42,6%) considerou o seu nível de capacitação como muito bom ou bom, e 56,8% conheciam a finalidade do fluoreto. A análise desse parâmetro foi realizada por 26 municípios (15,4%), dos quais 65,4% inseriam dados no Sistema de Informação de Vigilância da Qualidade da Água para Consumo Humano. Os motivos mais frequentes para não realizar a vigilância da fluoretação foram a não solicitação pela Secretaria de Estado da Saúde (36,1%) e a falta de material e de equipamentos (36,1%), de conhecimento (27,8%) e de profissionais capacitados (22,5%).

**Conclusão:**

As ações de vigilância da fluoretação não eram realizadas de forma sistemática na perspectiva da maioria dos técnicos dos municípios pesquisados. Diversas dificuldades e fragilidades foram apontadas, o que indica a necessidade de ações que apoiem o cumprimento do que está previsto na política nacional de vigilância da água para consumo humano.

**Aspectos éticos**

This research respected ethical principles, having obtained the following approval data:

**Research ethics committee**: **Opinion number**, **Approval date**

Universidade Federal de Goiás: 5.190.841, 31/12/2021

Secretaria de Estado de Saúde de Goiás: 5.249.236, 17/2/2022

Certificate of presentation of ethical assessment.: 53750321.9.0000.5083 53750321.9.3001.5082

**Informed consent record**: Obtained from all participants.

## Introdução

As doenças bucais representam um grande desafio para a saúde pública nos níveis global, nacional e local. A cárie dentária persiste com altos níveis de ocorrência no Brasil, apesar do declínio na dentição permanente de crianças e adolescentes (1). A fluoretação da água de abastecimento público consiste no ajuste do teor do íon fluoreto na água para consumo humano. Devido à sua efetividade, segurança, possibilidade de ampla cobertura e ao baixo custo, constitui o principal método coletivo de prevenção da cárie, sendo recomendada por instituições de pesquisa e por sistemas de saúde (2-4). 

A fluoretação é uma abordagem adotada de diferentes maneiras em todo o mundo. Enquanto em certos países ou regiões a aplicação dessa estratégia é extensiva, em outras, a sua implementação é limitada ou inexistente. No Brasil, a fluoretação teve início em 1953, em Baixo Guandu, Espírito Santo. Essa medida de saúde pública foi instituída pela Lei nº 6.050, de 24 de maio de 1974. 

Para garantir que a fluoretação resulte nos benefícios esperados e nos riscos mínimos para a população, é imprescindível verificar se os teores de fluoreto estão conforme os parâmetros estabelecidos para cada região. A vigilância do método contribui para que não haja uma oferta deficiente, que comprometa a prevenção da cárie, nem sobredosagem, que cause efeitos colaterais como a fluorose dentária (5). A implementação da fluoretação no país e a manutenção das ações de vigilância estão previstas na Política Nacional de Saúde Bucal (6), instituída pela Lei Federal nº 14.572, de 8 de maio de 2023 (7). 

O controle e a vigilância da qualidade da água destinada ao consumo humano são realizados no âmbito do Sistema Único de Saúde por órgãos de vigilância ambiental ou por organizações não diretamente responsáveis pela operação do sistema. Essas ações integram o Programa Nacional de Vigilância da Qualidade da Água para Consumo Humano e o seu respectivo Sistema de Informação de Vigilância da Qualidade da Água para Consumo Humano (Sisagua) (8). 

Os parâmetros para os teores de fluoreto são determinados em portarias do Ministério da Saúde (9,10). A concentração máxima deve ser de 1,5 mg/L ou ppm, podendo haver variações entre 0,6 e 1,7 mg/L, dependendo da média anual das temperaturas máximas diárias de cada localidade. 

Devido à falta de sistematização das informações sobre a implementação das ações de vigilância no país, pesquisas científicas podem contribuir para ampliar o conhecimento e apoiar a aplicação e a avaliação das políticas pertinentes. Estudos com essa finalidade são escassos, apesar de haver diversas publicações baseadas na análise laboratorial dos teores de fluoreto em nível local (11,12). Em 2005, cinco das 17 capitais brasileiras fluoretadas praticavam a vigilância da fluoretação (13). Nos municípios brasileiros com mais de 50 mil habitantes, foram reveladas desigualdades nas ações de vigilância entre as cinco macrorregiões brasileiras em 2014 (5,14). Na região Centro-Oeste, 68,6% dos municípios investigados possuíam água fluoretada, e apenas o Distrito Federal executava o heterocontrole em 2014 (15). 

Em Goiás, a fluoretação teve início em 1985, abrangendo, até 2023, 78,9% dos municípios. Dois estudos anteriores analisaram os teores de fluoreto em amostras de água de municípios goianos entre 2011 e 2013 (16) e em 2019 (17). As ações de vigilância da fluoretação no conjunto dos municípios fluoretados não é conhecida. Há, portanto, uma preocupante lacuna de conhecimento acerca dessa questão. Nesse sentido, os relatos dos profissionais diretamente envolvidos podem contribuir para elucidar aspectos que facilitam ou dificultam a realização da vigilância pelos municípios (18).

Este estudo teve como objetivo investigar as ações de vigilância da fluoretação da água de abastecimento público em Goiás, na perspectiva dos técnicos de vigilância ambiental das secretarias municipais de saúde. Os resultados podem ser úteis para revelar a situação atual e para subsidiar adequações na estratégia de vigilância da fluoretação da água no âmbito dos municípios e da Secretaria de Estado da Saúde, além de contribuírem para elucidar a situação em nível nacional.

## Métodos 

### Desenho do estudo

Trata-se de estudo observacional descritivo transversal. 

### Contexto 

Goiás é o estado mais populoso da região Centro-Oeste e o 11º do país. Sua população era de 7.056.495 habitantes e mais de 90% viviam em áreas urbanas, segundo o Censo de 2022 do IBGE. Dos 246 municípios de Goiás, 194 (78,9%) tinham água fluoretada em 2022 e foram incluídos no estudo. 

### Participantes 

Os participantes foram os profissionais (técnicos e fiscais em vigilância ambiental) das respectivas secretarias municipais de saúde responsáveis pela vigilância da qualidade da água de abastecimento público de cada município, o que totalizou 194 indivíduos. O convite para participar da pesquisa foi feito pela Superintendência de Vigilância em Saúde da Secretaria de Estado da Saúde. Para validar o critério de inclusão, o questionário incluía perguntas acerca das atribuições dos profissionais.

### Variáveis 

 As variáveis pesquisadas foram relacionadas às características sociodemográficas e ao trabalho dos técnicos; os conhecimentos e as percepções sobre a vigilância da fluoretação da água; os conhecimentos sobre a finalidade do fluoreto; os aspectos metodológicos da vigilância da fluoretação da água realizada pelos municípios; e os motivos para não realizar essa atividade e as perspectivas para a sua realização. 

### **Fontes de dados**/mensuração 

Para a coleta dos dados (junho de 2022 a junho de 2023), utilizou-se um questionário eletrônico autoaplicável, com perguntas fechadas e abertas, através da ferramenta Google Forms. O instrumento foi elaborado pelos pesquisadores com base nos objetivos da pesquisa e em estudos anteriores (5,11-14). A primeira versão foi revisada por dois docentes participantes da equipe do projeto. Em seguida, foi revista por gestores da Superintendência de Vigilância em Saúde da Secretaria de Estado da Saúde, responsáveis pela vigilância da qualidade da água de abastecimento público, bem como por quatro docentes da área da saúde bucal coletiva com expertise em questionários de pesquisa. 

A fase seguinte consistiu em um estudo-piloto, realizado com técnicos de cinco municípios por meio de reunião online. A versão final foi aplicada aos participantes em reunião online organizada para essa finalidade e convocada pela Superintendência de Vigilância em Saúde estadual. Os técnicos municipais que não participaram das reuniões receberam o questionário por e-mail posteriormente. 

### Métodos estatísticos

Os dados coletados por meio dos questionários foram armazenados automaticamente em uma planilha do software Microsoft Office Excel. Em seguida, foram analisados por meio de estatística descritiva (número e porcentagem) com o auxílio do software Statistical Package for the Social Sciences, versão 28.0. As respostas às questões abertas foram categorizadas por dois pesquisadores por meio da análise de conteúdo. As categorias emergentes foram quantificadas e submetidas à análise estatística descritiva.

## Resultados 

O questionário foi respondido por todos os técnicos convidados (n=194), sendo um de cada município fluoretado, totalizando 100,0% de taxa de resposta. Desses, 25 técnicos foram excluídos da análise, uma vez que informaram não exercer funções de vigilância direta da água de abastecimento. A idade dos participantes variou entre 19-63 anos (média 39,2; desvio-padrão 10,9) (Tabela 1). Pouco mais da metade era do sexo masculino (50,9%), tinha concluído ensino superior com ou sem pós-graduação (56,8%), era concursado (58,6%) e trabalhava há no máximo cinco anos nas secretarias municipais de saúde pesquisadas (56,2%). 

**Tabela 1:** Características sociodemográficas e relacionadas ao trabalho dos técnicos da vigilância da qualidade da água de abastecimento. Municípios fluoretados de Goiás, 2022-2023 (n=169)

Variáveis	n (%)
Sociodemográficas	
**Faixa etária** (anos)	
19-30	39 (23,1)
31-40	54 (32,0)
41+	68 (40,2)
Sem resposta	8 (4,7)
Sexo	
Feminino	82 (48,5)
Masculino	86 (50,9)
Outro	1 (0,6)
**Nível de escolaridade**	
Ensino fundamental	2 (1,2)
Ensino médio	71 (42,0)
Ensino superior	55 (32,5)
Ensino superior com pós-graduação	41 (24,3)
**Relacionadas ao trabalho na Secretaria Municipal de Saúde**	
**Tipo de vínculo atual**	
Concursado	99 (58,6)
Contratado	70 (41,4)
**Tempo de trabalho** (anos)	
Menos de um ano	23 (13,6)
1-5	72 (42,6)
6-10	34 (20,1)
11-20	36 (21,3)
21+	14 (8,3)

A maioria dos respondentes declarou conhecer parcialmente as normas do Ministério da Saúde (72,8%) e avaliou a análise do fluoreto como muito importante (56,2%) ou importante (31,4%) Tabela 2). Questionados sobre o nível de capacitação para atuarem na vigilância da fluoretação da água, 18,3% o avaliaram como muito bom, e 24,3%, como bom. 

**Tabela 2:** Conhecimento e percepção dos técnicos da vigilância da qualidade da água de abastecimento sobre a vigilância da fluoretação da água. Municípios fluoretados de Goiás, 2022-2023 (n=169)

Variáveis	n (%)
**Conhece as normas do Ministério da Saúde para o controle e a vigilância da qualidade da água?**	
Sim, totalmente	38 (22.5)
Sim, parcialmente	123 (72.8)
Não	8 (4.7)
**Como avalia a importância da análise do teor de fluoreto na água de abastecimento?**	
Muito importante	95 (56.2)
Importante	53 (31.4)
Indiferente	5 (3.0)
Pouco importante	0
Não sabe	16 (9.5)
**Como avalia seu nível de capacitação para atuar na vigilância da fluoretação da água?**	
Muito bom	31 (18.3)
Bom	41 (24.3)
Regular	49 (29.0)
Ruim	19 (11.2)
Péssimo	5 (3.0)
Não sabe	24 (14.2)

Do total de 169 respondentes, 56,8% relataram conhecer a finalidade do fluoreto na água de abastecimento (Tabela 3). Entre as respostas à pergunta aberta sobre a finalidade, as mais frequentes estiveram relacionadas à saúde bucal (82,3%). Nessa categoria, 61,4% mencionaram a prevenção, a redução ou o controle da cárie dentária. As respostas restantes referiram-se à saúde da boca ou dos dentes, à saúde geral e à qualidade da água. 

**Tabela 3:** Conhecimento dos técnicos da vigilância da qualidade da água de abastecimento sobre a finalidade do fluoreto. Municípios fluoretados de Goiás, 2022-2023 (n=169)

Variáveis	n (%)
**Sabe qual é a finalidade do fluoreto na água de abastecimento**	
Sim	96 (56,8)
Não	73 (43,2)
**Finalidade do fluoreto** (**n=96)a**	
Saúde bucal	79 (82,3)
Prevenção/redução/controle de cárie	58 (60,4)
Prevenção de cárie e fluorose	1 (1,0)
Saúde bucal	9 (9,4)
Formação/manutenção/fortalecimento/proteção dos dentes	9 (9,4)
Prevenção doenças bucais	1 (1,0)
Eliminação de algumas bactérias da boca	1 (1,0)
Saúde geral	5 (5,2)
Melhorias para a saúde	2 (2,1)
Prevenção de doenças	2 (2,1)
Prevenção e conscientização	1 (1,0)
Qualidade da água	7 (7,3)
Aumentar a qualidade da água	4 (4,2)
Avaliar a qualidade da água, se está com muito cloro	1 (1,0)
Detectar a pureza da água	1 (1,0)
“Fluoreto é veneno”	1 (1,0)
Não respondeu	5 (5,2)

^a^Pergunta aberta.

De acordo com os técnicos, 26 municípios (15,4%) analisaram o teor de fluoreto nos últimos cinco anos, enquanto 20,7% dos técnicos não souberam informar (Tabela 4). Dos 26 municípios que realizaram a análise, 88,5% tinham periodicidade mensal e 46,2% utilizaram o método fotométrico SPADNS. Os locais de realização das análises eram as secretarias municipais de saúde e o Laboratório Central de Saúde Pública, com predominância das primeiras (69,2%). Nas secretarias municipais de saúde (n=18), as análises eram feitas na maioria dos casos por profissionais treinados (88,9%) pela Secretaria de Estado da Saúde (56,2%). O apoio dessa secretaria estadual e das suas regionais de saúde foi referido por 38,5% dos municípios. Em 19,2% dos casos, a equipe de saúde bucal das secretarias municipais de saúde participou das ações de vigilância do fluoreto. Um total de 23,1% dos técnicos relatou dificuldades devido à falta de espaço, equipamentos, insumos, capacitação profissional e veículos para a coleta das amostras de água. A inserção dos dados referentes ao teor de fluoreto no Sisagua foi relatada por 65,4% dos respondentes.

**Tabela 4:** Aspectos metodológicos da vigilância da fluoretação da água realizada pelos municípios, de acordo com os técnicos da vigilância da qualidade da água de abastecimento. Municípios fluoretados de Goiás, 2022-2023

Variáveis	n (%)
**Análise do teor de fluoreto pelo município nos últimos cinco anos** (n=169)	
Sim	26 (15,4)
Não	108 (63,9)
Não sabe	35 (20,7)
**Periodicidade da análise** (n=26)	
Mensal	23 (88,5)
Semanal	1 (3,8)
Anual	1 (3,8)
Não sabe	1 (3,8)
**Método utilizado** (n=26)	
Fotométrico (SPADNS)	12 (46,2)
Colorimétrico (visual de alizarina)	6 (23,1)
Eletrométrico	3 (11,5)
Não sabe	5 (19,2)
**Local de realização da análise do fluoreto** (n=26)	
Secretaria Municipal de Saúde com equipamento portátil	18 (69,2)
Laboratório Central de Saúde Pública	5 (19,2)
Não sabe	3 (11,5)
**Treinamento do profissional, no caso das secretaria municipais de saúde** (n=18)	
Sim, pela Secretaria de Estado da Saúde	10 (55,6)
Sim, pela empresa que forneceu o equipamento	4 (22,2)
Sim, não sabe quem treinou	2 (11,1)
Não	2 (11,1)
**Apoio da Secretaria de Estado da Saúde e/ou das suas regionais de saúde para a análise do fluoreto** (n=26)	
Sim	10 (38,5)
Não	11 (42,3)
Não sabe	5 (19,2)
**Participação dos profissionais de saúde bucal** (n=26)	
Sim, da própria secretaria municipal de saúde	5 (19,2)
Não	16 (61,5)
Não sabe	5 (19,2)
**Dificuldades do município para a análise do fluoreto** (n=26)	
Falta de transporte/veículo para a coleta de água	2 (7,7)
Demora na aquisição dos produtos e na calibragem do equipamento	2 (7,7)
Falta de espaço adequado/equipamentos/capacitação do profissional	2 (7,7)
Nenhuma	18 (69,2)
Não sabe	2 (7,7)
**Inserção dos dados de vigilância no Sistema de Informação de Vigilância da Qualidade da Água para Consumo Humano** (n=26)	
Sim	17 (65,4)
Não	9 (34,6)

Observaram-se os resultados referentes aos 108 municípios que não realizaram a vigilância do fluoreto no período pesquisado (Tabela 5). Os motivos mais frequentes para não realizar essa ação foram a falta de obrigatoriedade/solicitação por parte da Secretaria de Estado da Saúde (36,1%), a falta de materiais e de equipamentos adequados (36,1%) e o desconhecimento por parte do município (27,8%). Um total de 58,3% dos respondentes manifestou a intenção de iniciar essa ação no futuro. Mais de um terço não sabia responder ou não respondeu às duas questões anteriores. Entre os que afirmaram interesse do município em realizar a vigilância no futuro (n=63), 55,6% informaram que necessitavam de apoio da Secretaria de Estado da Saúde para a aquisição de equipamentos e materiais adequados, suporte técnico e treinamento/capacitação dos profissionais. 

**Tabela 5:** Motivos para não realizar a vigilância da fluoretação e perspectivas para a sua realização, de acordo com os técnicos da vigilância da qualidade da água de abastecimento. Municípios fluoretados de Goiás, 2022-2023 (n=108^a^)

Variáveis	n (%)
**Motivos para não realizar a análise do fl^u^oretob**	
Não é obrigatório/não é solicitado pela Secretaria de Estado da Saúde	39 (36,1)
Falta de material e equipamentos adequados	30 (27,8)
O município não tem conhecimento sobre como realizar	24 (22,5)
Faltam profissionais capacitados	2 (1,9)
A empresa de tratamento da água realiza as análises	20 (18,5)
Não sabe	18 (16,7)
Sem resposta	39 (36,1)
**Intenção de realizar no futuro**	
Sim	63 (58,3)
Não	2 (1,9)
Não sabe	14 (13,0)
Sem resposta	29 (26,8)
**O que necessita da Secretaria de Estado da Saúdec** (n=63)	
Equipamento e materiais, suporte técnico e treinamento/capacitação	35 (55,6)
Equipamento e materiais somente	6 (9,5)
Capacitação somente	17 (27,0)
Não especificado	5 (7,9)

^a^Somente os municípios que não realizaram a vigilância do fluoreto; ^b^Mais de uma resposta, não totalizando 100,0%; ^c^Pergunta aberta.

## Discussão 

Este estudo buscou conhecer as ações de vigilância da fluoretação da água de abastecimento público em Goiás na perspectiva dos técnicos em vigilância ambiental das secretarias municipais de saúde. A ausência da análise do teor de fluoreto na água de abastecimento público na maioria dos municípios fluoretados revela uma preocupante lacuna na vigilância da qualidade da água, especialmente quando se considera a importância dessa medida para a saúde bucal da população (19). 

Apenas 26 municípios (15,4%) incluíam o fluoreto nas ações de vigilância. Nem todos o faziam com periodicidade mensal ou registravam os dados no Sisagua, em detrimento das normas estabelecidas pelo governo federal (9,10). Entre os 108 municípios que não realizaram a vigilância do fluoreto, 58,3% manifestaram interesse em iniciar essa ação. Evidencia-se, portanto, a necessidade de aprimorar a estratégia de vigilância da fluoretação da água no âmbito municipal e no estadual.

A baixa adesão às ações de vigilância do fluoreto foi observada também em municípios goianos com mais de 50 mil habitantes (14,15). Este estudo constitui a primeira investigação com abrangência estadual, incluindo uma ampla gama de variáveis. Na interpretação dos resultados, é importante considerar que esses se baseiam exclusivamente nas informações fornecidas pelos técnicos de cada município. Dessa forma, os relatos podem não ser exatos ou corresponder ao que é realizado na prática. O elevado percentual de ausência de respostas às questões específicas sobre o fluoreto pode indicar a baixa prioridade atribuída à vigilância desse parâmetro em detrimento dos demais, que são pactuados e acompanhados em conjunto com a Secretaria de Estado da Saúde.

A reduzida participação das equipes de saúde bucal nas ações de vigilância do fluoreto indica uma possível desconexão entre as estratégias de promoção da saúde bucal e as práticas de vigilância da qualidade da água nos municípios. Esses resultados mostram a importância da inclusão de conteúdos acerca da vigilância da fluoretação nos cursos de graduação em odontologia, os quais podem contribuir para a integração das equipes de saúde bucal e de vigilância na perspectiva dos fatores de risco comuns às doenças bucais e sistêmicas (20).

O predomínio do nível mais alto de escolaridade e de admissão por concurso público podem ser considerados aspectos positivos para o desempenho das atividades de vigilância. Em contrapartida, a exemplo do nível de comprometimento organizacional de fiscais da vigilância sanitária de municípios de Goiás, houve maior comprometimento afetivo e maior sentimento de obrigação no desempenho laboral entre os respondentes com idade mais avançada, com menor escolaridade e sem vínculo efetivo com as secretarias municipais de saúde (21).

Um elevado percentual de respondentes do total da amostra considerou a análise do fluoreto como muito importante e importante, o que sugere o reconhecimento da relevância dessa estratégia para a saúde bucal da população. No entanto, menos da metade avaliou positivamente o nível de capacitação para essa atividade. Pouco mais da metade relatou conhecer a finalidade específica do fluoreto, dos quais quase 18% não mencionaram quaisquer benefícios à saúde bucal. Destaca-se o relato isolado de um técnico que classificou o fluoreto como veneno, o que indica a existência de desinformação acerca dessa substância, inclusive entre profissionais do Sistema Único de Saúde. 

Há lacunas preocupantes no que se refere aos conhecimentos técnicos dos participantes, o que pode gerar impacto negativo no desempenho das suas funções. Recomendam-se estratégias de educação permanente em saúde (22) com base em evidências científicas de qualidade para que os técnicos possam realizar as suas atividades em conformidade com a legislação (9-10) e garantir a qualidade da água disponibilizada à população. Como exemplo de estratégia, destaca-se a inclusão de módulos específicos sobre fluoretação (2,3) em atividades de capacitação ou de atualização no âmbito do Programa Nacional de Vigilância da Água para Consumo Humano (8). 

A falta de materiais, equipamentos, transportes, conhecimentos e profissionais capacitados, conforme relatado, constitui barreiras à análise do fluoreto pelos municípios e é um ponto crucial a ser considerado. Resultados semelhantes foram relatados em um trabalho que avaliou as dificuldades e as limitações enfrentadas pelos técnicos responsáveis pela vigilância da qualidade da água em sete municípios da região metropolitana da grande Vitória, Espírito Santo (25). A falta de recursos essenciais pode comprometer a capacidade dos municípios para realizar análises precisas e consistentes, prejudicando a efetividade da fluoretação da água. Neste estudo, os técnicos dos municípios que não realizavam a vigilância da fluoretação indicaram também a falta de obrigatoriedade e de solicitação por parte da Secretaria de Estado da Saúde. 

A subutilização do Sisagua para o registro de dados de vigilância do fluoreto tem sido relatada nos níveis nacional e local (13,23-25). Neste estudo, 65,4% dos 26 municípios goianos que realizavam a vigilância do fluoreto informaram inserir os respectivos dados no sistema. Essa situação mostra a necessidade de monitoramento dessas ações no nível municipal por parte das secretarias estaduais de saúde, buscando investigar inconsistências e possíveis erros, bem como oferecer suporte técnico quando necessário.

Embora a fluoretação da água de abastecimento público esteja prevista na legislação federal desde 1974 e seja respaldada por ampla evidência científica quanto à sua efetividade (3,4), a vigilância desse método enfrenta importantes desafios operacionais, estruturais e políticos nos municípios brasileiros. Essa realidade reflete uma dificuldade recorrente no setor da saúde pública: a fragilidade na implementação das políticas públicas. A etapa de implementação é frequentemente desvalorizada e marcada por entraves em nível local, no qual os conflitos de interesse, a ausência de recursos técnicos e humanos e a baixa prioridade política prejudicam a efetivação das diretrizes nacionais (26).

No campo específico da vigilância em saúde, a consolidação da política nacional esbarra na insuficiência de sistemas de informação, na baixa integração intersetorial e na fragmentação entre vigilância e atenção básica, o que compromete a efetividade das ações (27). Tais dificuldades são ainda mais acentuadas em políticas como a vigilância da fluoretação, cuja natureza intersetorial exige cooperação entre os setores do saneamento, da saúde ambiental e da saúde bucal.

No que se refere à saúde bucal, apesar dos avanços normativos e da institucionalização de práticas de vigilância (6,7), persistem desafios relacionados à escassez de profissionais qualificados, à fragilidade dos sistemas locais de monitoramento e à ausência de incentivos para a continuidade das ações (28). A coordenação federativa das políticas públicas de saúde no Brasil ainda carece de mecanismos efetivos de indução e de responsabilização entre os entes federados para a uniformização de práticas e o cumprimento das normas estabelecidas (29).

Para compreender a fraca adesão à vigilância da fluoretação, é importante reconhecer que a simples existência de diretrizes legais não garante a sua efetivação. A implementação é um processo político-institucional complexo, que depende de capacidade técnica, de apoio político local e de uma estrutura administrativa adequada para ser concretizado. 

Como ponto forte deste estudo, destacou-se o fato de a investigação ter sido feita a partir da visão dos trabalhadores da vigilância da água no Sistema Único de Saúde, que revelaram aspectos não identificados em pesquisas laboratoriais ou com base nos sistemas de informação. Outros pontos fortes foram a representatividade no nível estadual da amostra e a abrangência das variáveis investigadas. 

Como principais limitações do estudo, consideraram-se a baixa confiabilidade de dados obtidos em questionários por via eletrônica, de forma geral (30), e a não realização de teste de confiabilidade do instrumento. Existe, ainda, a possibilidade de viés de opinião, que pode ter conduzido a respostas tendenciosas e a resultados que não refletiam a verdadeira opinião da população estudada. 

Com base nos relatos dos técnicos dos municípios pesquisados, conclui-se que as ações de vigilância da fluoretação não eram realizadas de forma sistemática. Apenas 15,4% dos municípios realizavam monitoramento, dos quais 65,4% inseriam dados no Sisagua. Os principais motivos para não realizar a análise do fluoreto foram relacionados à falta de conhecimento e de capacitação (50,3%), à falta de material e de equipamentos (36,1%) e a não obrigatoriedade da ação (36,1%). 

As dificuldades e as fragilidades encontradas indicam a necessidade de ações no âmbito dos municípios e das secretarias de estado da saúde que apoiem o cumprimento do que está previsto na política nacional de vigilância da água e nas respectivas normas (8-10). A inclusão do fluoreto entre os parâmetros a serem monitorados é imprescindível para garantir a efetividade e a segurança da fluoretação na prevenção da cárie dentária na população (7). 

## Data Availability

O banco de dados e os códigos de análise utilizados na pesquisa estarão disponíveis mediante solicitação aos autores através do contato informado na seção Correspondência.
